# Supplementary material for: Characterization of the Protective Immune Responses Conferred by Recombinant BCG Overexpressing Components of Mycobacterium tuberculosis Sec Protein Export System
Source: Vaccines (Basel). 2022 Jun 14;10(6):945. doi: 10.3390/vaccines10060945 (PMC9229301; doi:10.3390/vaccines10060945)
Supplement: Supplementary file 1 [file vaccines-10-00945-s001.zip › vaccines-1740152-supplementary.pdf]

vaccines

MDPI

SUPPLEMENTARY INFORMATION

Article

Characterization of the Protective Immune Responses Conferred by Recombinant BCG Overexpressing Components of *Mycobacterium tuberculosis* Sec Protein Export System

Annuurun Nisa [1,†,‡], Claudio Counoupas [1,2,3,‡], Rachel Pinto [1], Warwick J. Britton [2,4] and James A. Triccas [1,3,*]

[1] School of Medical Sciences, Faculty of Medicine and Health, The University of Sydney, Camperdown, NSW 2006, Australia; a.nisa@rutgers.edu (A.N.); c.counoupas@centenary.org.au (C.C.); rachel.pinto@sydney.edu.au (R.P.)
[2] Tuberculosis Research Program at the Centenary Institute, The University of Sydney, Sydney, NSW 2006, Australia; w.britton@centenary.org.au
[3] Sydney Institute for Infectious Diseases and the Charles Perkins Centre, The University of Sydney, Camperdown, NSW 2006, Australia
[4] Department of Clinical Immunology, Royal Prince Alfred Hospital, Camperdown, NSW 2005, Australia
* Correspondence: jamie.triccas@sydney.edu.au
† Present address: Public Health Research Institute, New Jersey Medical School, Rutgers University, Newark, NJ 07103, USA.
‡ These authors contributed equally to this work.

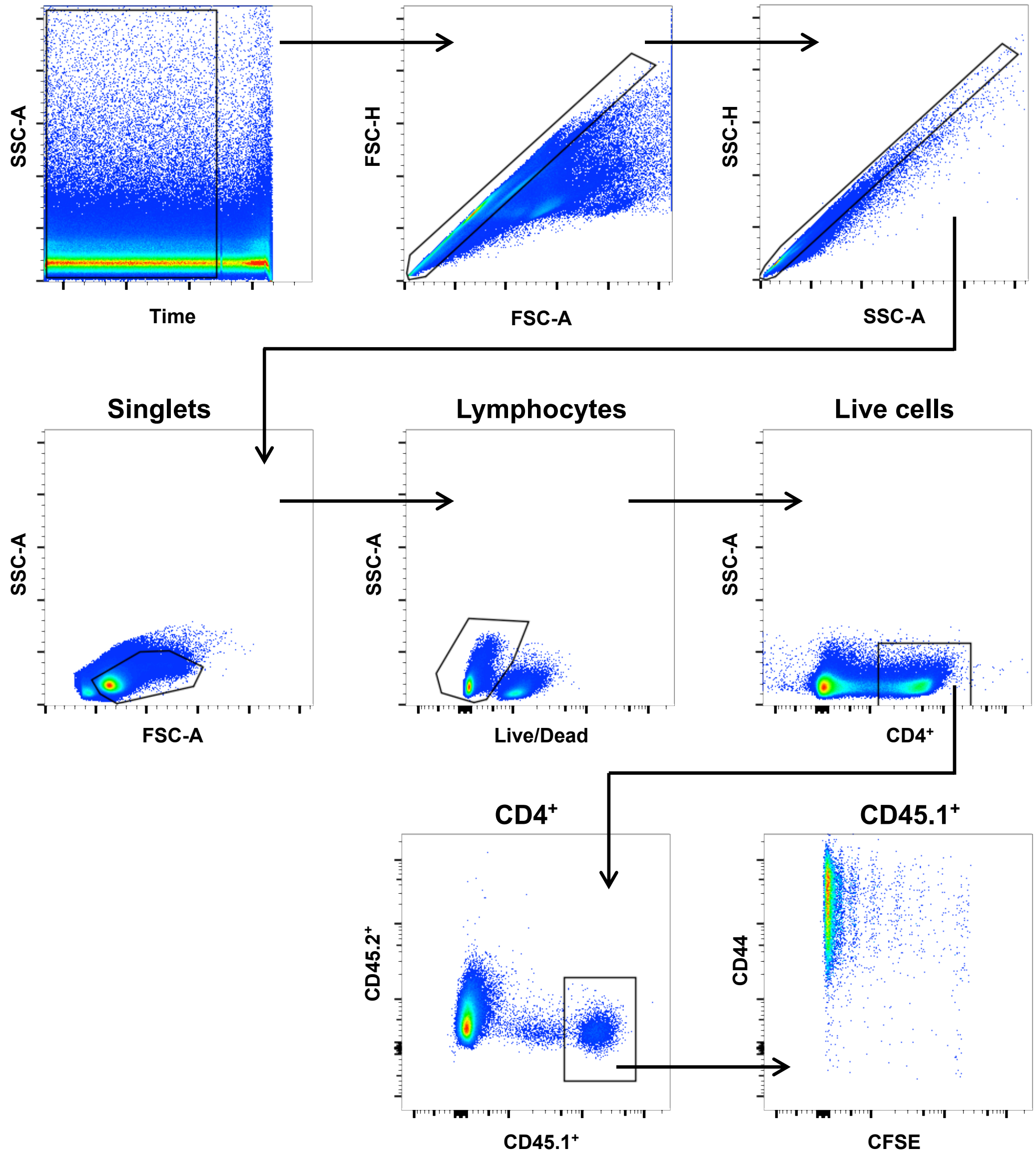

Figure S1. Gating strategy used for assessing p25-specific CD4⁺ T cells proliferation following vaccination. A time gate was applied to verify consistent fluorescence signal during sample acquisition. After excluding debris by FSC and SSC, dead cells were excluded by live/dead UV stain. The CD45.2⁺ and CD45.1⁺ T cell populations were gated from live CD4⁺ T cell population. The frequencies of activated proliferating p25-specific CD45.1⁺ T cells were then assessed by looking at the expression of CD44 and CFSElo/int/hi.

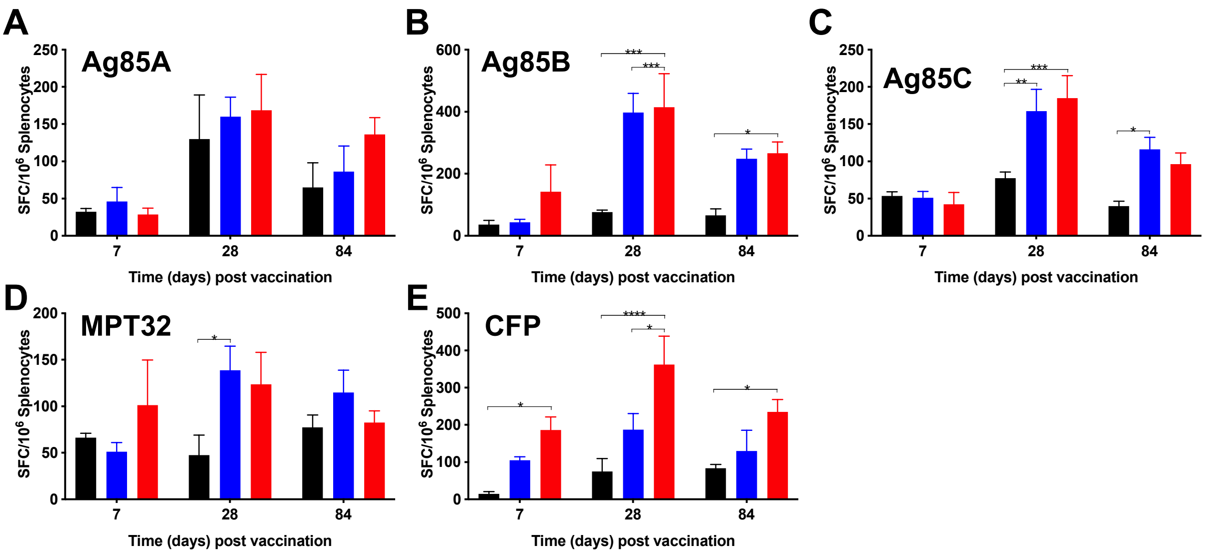

Figure S2. Induction of antigen specific IFNγ-secreting cells in mouse lymph nodes following BCG^{SecDFG} vaccination. C57BL/6 mice were vaccinated as in Figure 2. Lymph nodes cells from day 7, 28 and 84 were stimulated *ex vivo* with 10 µg/mL of Ag85A (A), Ag85B (B), Ag85C (C), MPT32 (D) or CFP (E) for approximately 20 hours at 37° C. The number of antigen-specific IFNγ-secreting cells were enumerated by ELISPOT. The data are the means ± SEM and representative of two independent experiments. Statistical significance was determined by ANOVA with Tukey's multiple comparisons test (* $p<0.1$; ** $p<0.01$).

Table S1. Flow cytometry antibodies used for surface staining.

Marker	Fluorophore	Clone	Dilution	Company	Cat. #
Live/Dead	Blue		1:300	ThermoFisher	L23105
Fc Block	purified	2462	1:300	BD	553141
CD4	AF700	RM414	1:200	BD	557956
CD44	BV605	IM7	1:300	BD	563058
CD8	APC-Cy7	53-6.7	1:200	BD	557654
CXCR3	BV605	CXCR3-173	1:100	BioLegend	551960
CD45.1	Pacific Blue	A20	1:200	BioLegend	110722
CD45.2	BV510	104	1:200	BioLegend	109838
CD62L	eFluor450	DREG-56	1:200	ThermoFisher	48-0621-82

Table S2. Flow cytometry antibodies used for intracellular staining.

Marker	Fluorophore	Clone	Dilution	Company	Cat. #
IFN-γ	PECy7	XMG1-2	1:200	BD	557649
IL-17	PB	TC11-18H10.1	1:200	BioLegend	506918
IL-2	PE	JES6-5H4	1:200	BD	554428
TNF	PerCP-Cy5.5	MP6-XT22	1:200	BD	560659
